# G-quadruplexes in viruses: function and potential therapeutic applications

**DOI:** 10.1093/nar/gku999

**Published:** 2014-10-20

**Authors:** Mathieu Métifiot, Samir Amrane, Simon Litvak, Marie-Line Andreola

**Affiliations:** 1 CNRS UMR-5234, Université de Bordeaux, 146 Rue Léo Saignat, 33076 Bordeaux, France; 2 INSERM, U869, IECB, ARNA laboratory, Université de Bordeaux, 2 Rue Robert Escarpit 33600 Pessac, France

## Abstract

G-rich nucleic acids can form non-canonical G-quadruplex structures (G4s) in which four guanines fold in a planar arrangement through Hoogsteen hydrogen bonds. Although many biochemical and structural studies have focused on DNA sequences containing successive, adjacent guanines that spontaneously fold into G4s, evidence for their *in vivo* relevance has recently begun to accumulate. Complete sequencing of the human genome highlighted the presence of ∼300 000 sequences that can potentially form G4s. Likewise, the presence of putative G4-sequences has been reported in various viruses genomes [e.g., Human immunodeficiency virus (HIV-1), Epstein–Barr virus (EBV), papillomavirus (HPV)]. Many studies have focused on telomeric G4s and how their dynamics are regulated to enable telomere synthesis. Moreover, a role for G4s has been proposed in cellular and viral replication, recombination and gene expression control. In parallel, DNA aptamers that form G4s have been described as inhibitors and diagnostic tools to detect viruses [e.g., hepatitis A virus (HAV), EBV, cauliflower mosaic virus (CaMV), severe acute respiratory syndrome virus (SARS), simian virus 40 (SV40)]. Here, special emphasis will be given to the possible role of these structures in a virus life cycle as well as the use of G4-forming oligonucleotides as potential antiviral agents and innovative tools.

## FROM TETRADS OF GUANOSINE TO G-QUADRUPLEXES: DISCOVERY AND TOPOLOGY

**Figure 1.**
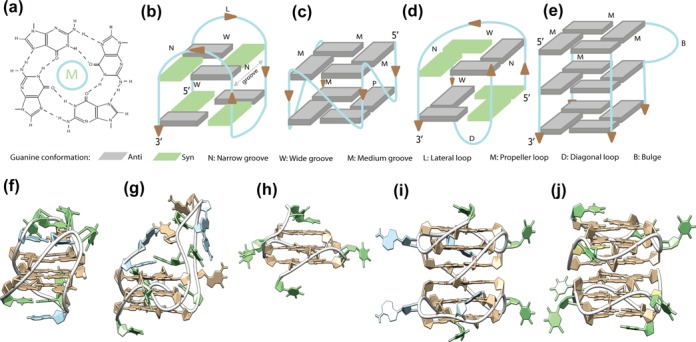
(**a**–**e**) Schematic representation of G4 topologies. (a) A guanine tetrad stabilized by eight Hoogsteen hydrogen bonds and a central monovalent cation (M). (b) Intramolecular antiparallel G4 topology with two tetrads, wide and narrow grooves and only lateral loops. (c) Intramolecular parallel G4 topology with two tetrads, medium grooves and only propeller loops. (d) Dimeric antiparallel G4 topology with two tetrads, wide and narrow grooves and diagonal loops. (e) Tetramolecular parallel G4 topology with three tetrads, only medium grooves and no loops. (**f**–**j**) Examples of G4 structures. (f) Intramolecular anti-parallel G4 structure with two tetrads for the telomeric sequence (PDB ID: 2KF8). (g) Intramolecular parallel G4 structure with three tetrads and a nine nucleotide central loop for the human CEB25 mini-satellite sequence (PDB ID: 2LPW). (h) Intramolecular parallel G4 structure with three tetrads for the T30177 anti-HIV aptamer (PDB ID: 2M4P). (i) Interlocked bimolecular parallel G4 structure with six tetrads for the 93del anti-HIV aptamer (PDB ID: 1Y8D). (j) Two stacked parallel G4 structures with three tetrads each observed for the T30923 anti-HIV aptamer (PDB ID: 2LE6).

Almost a century ago, the ability of guanosine, but not guanine, to form viscous gels was described (1). Fifty years later, X-ray diffraction data clearly showed that the guanosine moieties in these gels were arranged in a tetrameric organization linked by eight Hoogsteen hydrogen bonds (Figure 1) (2,3). These hydrogen bonds differ from the bonds observed in canonical Watson–Crick pairing and involve the interaction of the N7 group from one guanine with the exocyclic amino group from a neighboring base (Figure 1a). Therefore, a G-tetrad or a G-quartet results from planar association between four guanines that are held together by eight hydrogen bonds and coordinated with a central Na+ or K+ cation (4–8). In addition, nucleoside derivatives were also used to confirm the structural properties of G-quartets (9–14).

Conversely, a G-quadruplex or G4 is formed by nucleic acid sequences (DNA or RNA) containing G-tracts or G-blocks (adjacent runs of guanines) and composed of various numbers of guanines. Depending on the nucleotide sequence, the way G4s can be formed presents a high degree of diversity. The core of a G4 is based on stacking between two or more G-tetrads, wherein the guanines can adopt either a *syn* or an *anti* glycosidic bond angle conformation. Consequently, each of the four G-tracts that form the core of the structure can run in the same or opposite direction with respect to its two neighbors, forming parallel, anti-parallel or hybrid core conformations. Depending on these orientations, the G-blocks delimit four negatively charged grooves of different sizes: narrow, medium or wide (Figure 1b–e). For intra-molecular structures (Figure 1b and c), the four G-tracts belong to the same oligonucleotide and are attached by linkers with variable nucleotide sequences and lengths. These loops can adopt three different conformations: lateral, diagonal or propeller (Figure 1b–d). The bi- or tetra-molecular G4 structures (Figure 1d-e) are assembled from G-tracts belonging to two or four different strands. The G-blocks can also be interrupted by one to seven non-G nucleotides, which result in bulges that protrude from the G4 core (Figure 1e). In contrast to the almost mono-morphic canonical duplex, these variable structural parameters are directly related to the nucleotide primary sequence. This unique family of globular-shaped nucleic acid structures (Figure 1f–j) presents a high level of plasticity that enables various applications (see ‘G4s as antiviral agents’ section).

However, a potential physiological role for G4s has remained controversial for many years. Nonetheless, interest in G-quaduplexes has increased over time, with thousands of reports and reviews published on several aspects of G4s, including the biophysical and chemical characteristics as well as biological function in prokaryotes and eukaryotes (5,15–24). With an increasing volume of sequencing data, databases and algorithms have also been developed to enable search and mapping of G4 in a few mammalians as well as hundreds of bacterial species (25–28).

Additionally, G4s present an intrinsic resistance to ‘regular’ nucleases. However, G4 specific nucleases have been isolated, first in yeast with KEM1/SEP1 (29) and later in humans with GQN1 (30). Later, a DNA binding protein (G4R1/RHAU) that can unfold RNA/DNA quadruplexes was isolated in human cells (31,32), which further highlights the biological relevance of G4s. Finally, development of G4-specific probes such as monoclonal antibodies (33–35) and small chemical ligands (36–41) facilitated *in vivo* studies that support quadruplex formation in cells. Taken together, G4s are likely present in several significant genomic regions and may be a key component in important cellular processes (42,43).

## BIOLOGICAL ROLES OF G4S IN THE GENOME

The sequencing of the human genome highlighted the presence of many sequences enriched in guanines that can potentially form quadruplexes [for a review, see (44)]. In theory, more than 350 000 putative G-tetrads may be formed with loops of 1–7 nucleotides, and over 700 000, with loops of up to 12 nucleotides. However, the genomic DNA is dynamic, and quadruplexes, duplexes or other structural forms at those sites will be influenced by chromatin and other DNA-binding proteins. Thus, G4 formation depends on the cell type and cell cycle, ultimately impacted by environmental conditions and stresses.

### The telomeres

Without clear signaling and tight regulation, extremities present in linear chromosomes could be recognized as damage DNA and it would be deleterious for the cell if processed as such by repair mechanisms (45). Telomere length has also been linked to the lifespan of organisms. Telomeres are nucleoprotein structures found at the end of chromosomes protecting the genome from instability. This terminal region of chromosomes contains long tracts (several kilobases) of double stranded TTAGGG:CCCTAA repeats ending with a 3′ protrusion of single stranded TTAGGG repeats (10–50 repeats). Telomerase is a ribonucleoprotein enzyme that adds TTAGGG repeats to the 3′ end of DNA strands in the telomere regions. Human telomerase is a heterodimeric complex composed of (i) telomerase reverse transcriptase (TERT), a DNA-polymerase RNA-dependent, (ii) a single-stranded RNA template referred to as telomerase RNA component (TERC) and (iii) dyskerin [dyskeratosis congenita 1 (DKC1)], a pseudouridine synthase binding TERC through the H/ACA motif and stabilizing the telomerase complex (46,47).

**Figure 2.**
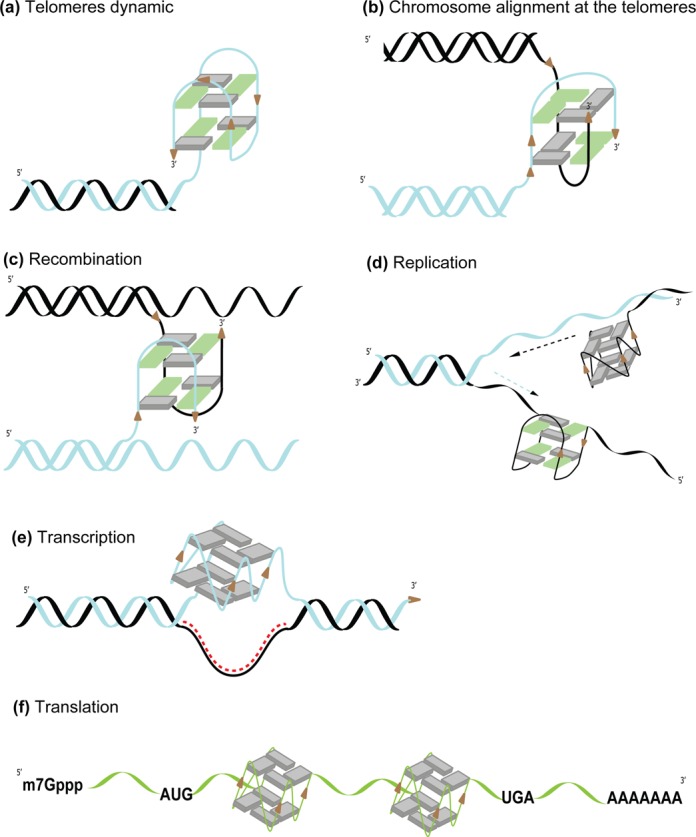
Role of G4 structures in the genome. (**a**) Formation of a G4 at the 3′ end of a chromosome. (**b**) Formation of an intermolecular G4 between two telomeres allows chromosomes’ alignment. (**c**) Intermolecular G4 formation leads to physical crossing and potential recombination sites at different locations of the DNA (**d**) Collision of the replication fork with G4s formed on the leading and lagging DNA strands. (**e**) Transcription can lead to the formation of a DNA/RNA duplex (R-loop) on the template strand and formation of a G4 (G-loop) on the non-template strand. (**f**) Formation of G4s within mRNAs block translation, as observed for EBNA1 coding mRNA (131).

Accumulated experimental data indicate the presence of G4s in telomeric DNA (6,48–52). For example, DNA2, a helicase/nuclease that cleaves G4s, is involved in maintaining telomere integrity (53,54). Altogether, these observations clearly establish that telomeric G4s are crucial structures for regulating telomere maintenance, thus providing a mechanism for controlling cell proliferation (55,56). At the 3′ ends of telomeres, if the G-rich overhang is longer than four TTAGGG repeats (>23 nucleotides), it can fold over itself and form secondary structures, including G4s (Figure 2a). Those structures prevent telomere elongation by the telomerase complex (57). Accordingly, small G4-ligands (e.g. telomestatin) and G4-binding proteins (e.g. TRF2) show anti-proliferative and potential anti-tumor activities through telomere interference (36,58–61). However, the exact mechanism is more complicated as telomestatin derivatives present telomerase independent activity, most likely through targeting G4s involved in tumor growth elsewhere in the genome (62–64).

### DNA replication

DNA replication is highly regulated, which ensures faithful reproduction of genetic information through each cell cycle. In eukaryotes, this process is initiated at thousands of DNA replication origins (ORIs) distributed along each chromosome (65). Initially, a pre-replicative complex (Pre-RC) is assembled during late mitosis and the early G1 phase; it evolves during the S phase to a pre-initiation complex through initiator protein activity (e.g., cyclin‐dependent kinases, Cdc7, Cdc45 and minichromosome maintenance complex (MCM) proteins). This step involves unwinding the origin and DNA polymerase recruitment. A tissue-specific temporal system exists, and only a few origins undergo ‘firing’ (replication fork elongation). The remaining origins are dormant backups used under stress (e.g., DNA damage and collisions with transcription machinery). Studies on organisms ranging from drosophila to humans have highlighted that repeated elements are enriched with G residues (66). *In vitro*, origin G-rich repeated element (OGRE) sequences form G4s, and point mutations that affect the stability of these G4s also impair origin function. Even if a 200-bp *cis*-regulatory element is necessary for efficient initiation, G4 structure formation at ORIs might be the key to selecting the firing origins (67,68). Additionally, G4s formed in the lagging strand can lead to the stalling of the replication fork (Figure 2d). On the other side, formation of G4s in the leading strand can displaced it from the template and causes polymerase slippage (Figure 2d). Such events could participate to the unusual expansions of G4 forming mini-satellite sequences (69).

### Gene expression

Altered gene expression levels are implicated in many human diseases. G4s have been found in the promoter region of many genes and implicated in transcription and protein translation regulation [for a review, see (70)]. Particularly interesting, the P1 region, which is upstream of the c-myc promoter, is highly sensitive to DNase I and S1 nucleases (nuclease-hypersensitive element or NHE). This purine-rich sequence can form an intramolecular G4 (71–74), which structure has been determined by nuclear magnetic resonance (NMR) (75). This NHEIII_1_ region controls up to 90% of gene transcription activation, and the G4 acts as a transcriptional repressor element (76,77). Because interactions between DNA-binding proteins and local DNA supercoiling can impact the equilibrium between duplex/single-stranded DNA (active transcription) and quadruplex DNA (silent), c-myc expression and, ultimately, cell proliferation may be modulated. Nucleolin (a nucleolar phosphoprotein) and ADAR1 (a Z-DNA binding/RNA editing protein) both bind the c-myc promoter *in vivo* and stabilize the G4 structure of the promoter (78,79).

Due to the high therapeutic potential of genomic G4s, studies have also been performed on promoters of other genes implicated in cancer biology. For example, KRAS is one of the most frequently mutated genes in human cancer. Its promoter contains a G-rich nuclease hypersensitive element that is critical for transcription (80). The polypurine strand forms G4 structures that bind crucial DNA repair proteins (e.g., PARP-1, Ku70) (81). Direct evidence for G4 formation was also reported for the proximal promoter region of the RET proto-oncogene, and targeting this region with a small molecule represses RET proto-oncogene transcription (82).

Although many G-rich sequences have been identified in the genome, they enable generation of corresponding G-rich RNAs when the sequences are located in transcription units. Ultimately, these RNAs can fold into quadruplex structures and impact gene expression (83). In 5′-untranslated regions (5′-UTRs), G4s can act as translation repressors (84–86). G4 structures have also been proposed to regulate translation driven by IRES domains in the absence of an mRNA cap [for a recent review, see (87) and the references therein]. Finally, it has recently been shown that G4 RNAs in coding regions can stimulate ribosomal frame-shifts *in vitro* and in cultured cells providing a role for G4 in translational regulation (88,89).

### Genome stability

A major challenge for future studies is to better understand genome instability, cancer and severe neurological/neuromuscular defects origins. Repeated sequences, such as polynucleotide repeats (three and above), mini- and mega-satellites, are source of genome instability through either expansion or contraction (90). However, DNA secondary structures associated with those tandem repeats may be crucial elements in the expansion phenomenon. For progressive myoclonus epilepsy type-1 (EPM1), expansion of the dodecamer sequence (CGCG_4_CG_4_) within the cystatin B promoter is thought to depend on G4 formation (91).

In addition to this potential deleterious consequence, G4s have also been implicated in development of specific immune responses. In antigen-activated B cells, class switch recombination promotes deletion of a DNA fragment (several kilobases) that joins a new constant region to the variable immunoglobulin chain (92). These switch regions (S regions) are intronic, G-rich and repetitive sequences that form G-loops upon transcription (Figure 2e). *In vivo*, class switch recombination depends on the activity of AID (a cytidine deaminase) and MutSα (MSH2/MSH6, and conserved DNA repair factors involved in mismatch repair). AID promotes creation of U:G mismatches, and MutSα binds with high affinity the G4 DNA formed upon transcription of the S regions.

In parallel, pathogens have also developed counter-measures to evade the immune system. The role of G-quartets in this evasion is related to antigenic variation. For *Plasmodium falciparum*, G-rich sequences have been found in the upstream region of group B *var* genes that can form *in vitro* stable G4s (93). Similarly, a G4-forming sequence (5′-G_3_TG_3_TTG_3_TG_3_) is located upstream of the *pilE* expression locus in *Neisseria gonorrhoeae* (94). Formation of G4s is necessary for initiating this pilin antigenic variation through recombination, and interactions with RecA could facilitate this specialized reaction. Overall, immune evasion and immunoglobulin gene class switch recombination in vertebrates are clearly analogous mechanistically (95).

## THE ROLE OF G4S IN THE VIRUS LIFE CYCLE

### Human immunodeficiency virus

Searches for G4s and elucidation of their function in the viruses’ genome have mainly focused on the human immunodeficiency virus (HIV), which causes acquired immunodeficiency syndrome (AIDS). Currently, ∼35 million people are infected with HIV worldwide. With over 2 million new infections and ∼1.6 million deaths from AIDS per year, the pandemic continues to spread. Even without a vaccine, development of highly active anti-retroviral therapies have allowed people to live with HIV as a chronic disease (96). However, viruses within T cells remain fully capable of replicating and infecting other cells if the drug pressure is removed or when resistance emerges. Thus, new drugs must be developed to overcome the treatment's genetic barrier.

HIV-1 is an RNA virus in the *Lentivirus* genus and is part of the *Retroviridae* family. Lentiviruses are single-stranded, positive-sense, enveloped RNA viruses. HIV-1 particles contain two molecules of genomic RNA that are converted into double-stranded DNA by the viral reverse transcriptase (RT). The resulting viral DNA is then imported into the nucleus and insertion into the cellular DNA is catalyzed by the virally encoded integrase (IN). Once integrated, transcription from the viral promoter at the 5′ long terminal repeat generates mRNAs that code various viral proteins and genomic RNA. Alternatively, the provirus may become latent, which allows the virus and its host cell to avoid detection by the immune system. The presence of G4 structures has been highlighted at both RNA and DNA levels with implications throughout the viral life cycle.

### HIV-1 RNA dimerization and recombination

Retroviral RNAs dimerize in the cytoplasm of an infected cell allowing two copies of the genome to be encapsidated in the newly produced virion (97). While a single copy of the genome is sufficient for viral replication, the second copy is also used during reverse transcription, and the viral RT switches multiple times between the two RNA molecules (98,99). The strand transfers are partially responsible for the viral variability through production of recombinant molecules. Therefore, understanding the mechanisms that drive dimerization and recombination is essential.

Dimerization is a two-step process that involves sequences upstream of the splice donor site (100,101). The sequences involved in initial dimerization and encapsidation partially overlap at the 5′ end of the viral genome. One of the sequences is a highly conserved dimer initiation site (DIS) that forms a stem loop. A concentration-dependent kissing–loop interaction is initiated from contacts between consecutive guanines (102); the interaction then spreads to the stems. However, this interaction does not seem sufficiently strong to keep the two copies together during reverse transcription.

Several studies have identified a G-rich sequence that form bi-molecular G4 structures in the *gag* region of the HIV-1 genome, near the DIS (103–105). Localization of these G4-forming sequences correlates with recombination hot spots and exhibits an increased rate of template switching that highlights a potential role for these structures (106). Supporting this hypothesis, recombination in the U3 domain is cation-dependent and is lower in the presence of Li^+^, which is a metal ion that fails to stabilize G4s (106).

Short RNA templates from the central region of the HIV-1 genome contain G-rich sequences near the central poly-purine tract (cPPT) at the 3′ end of the *pol* gene (IN coding sequence); this is a region where one of the two primers used for synthesizing the (−) strand DNA is produced during reverse transcription. These sequences can form both intramolecular and dimeric G4 structures. Moreover, reconstituted systems have confirmed that G4 structures near the cPPT facilitate strand transfer and promote template switching by the RT (107). Interestingly, certain elements from the cPPT region are involved in forming the cPPT flap, which is a region that plays an important role in nuclear entry of the double-stranded DNA (108,109). Taken together, the G-rich regions located at the 5′ end of the genome and in the central region are likely maintained in proximity through inter-RNA G4 formation with a crucial role in HIV-1 replication.

### HIV-1 nucleocapsid: G4 chaperone properties

**Figure 3.**
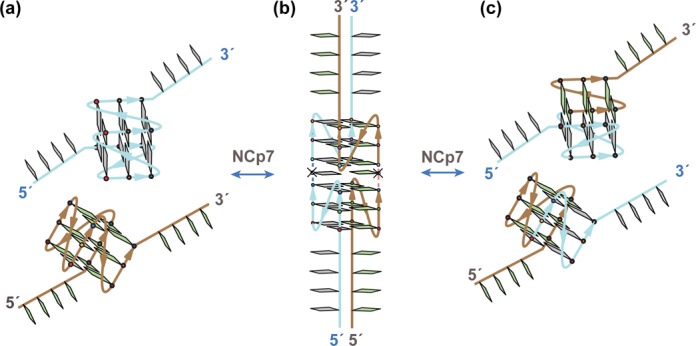
Model of viral RNA:RNA dimerization and recombination involving a pair of monomeric G4s mediated by a synaptic dimeric G4 intermediate [inspired and adapted from a model of intergenic recombination (115)]. (**a**) A pair of parallel-stranded monomeric G4s. (**b**) NCp7 chaperon properties favor formation of an inter-RNA synaptic intermediate involving a parallel-stranded dimeric G4. Cleavage, rotation and subsequent rejoining are mediated by a potential, undefined nuclease-topoisomerase-like enzyme complex. (**c**) Following dimerization and/or recombination, NCp7 annealing properties favor formation of a new pair of parallel-stranded monomeric G4s.

Retroviral nucleocapsid proteins (NCp) are multifunctional elements encoded in the *gag* gene. Notably, NCp participate in many retroviral cycle steps by remodeling nucleic acid structures to favor thermodynamically stable conformations. They are referred to as nucleic acid chaperones and interact with nucleic acid phosphodiester backbones through electrostatic interactions thanks to basic residues (especially residues in the N-terminus) [for reviews, see (110,111)]. Moreover, HIV-1 NCp (NCp7) exhibits sequence-specific binding to runs of Gs, UGs or TGs through interactions involving its two zinc fingers (CCHC motifs separated by a proline-rich linker). Although there is no doubt that NCp7 tightly binds G4 sequences, data in the literature shows that a hydrophobic interaction engaged by the C-terminal zinc finger of the protein may lead to G4 stabilization (112), while high concentration of NCp7 promotes G4 unfolding (113). A recent biophysical study using high-speed atomic force microscopy (HS-AFM) addressed direct and real-time investigations on the molecular chaperone activity at the single-molecule level (114). NCp7 can efficiently promote bimolecular G4 formation and is able to anneal the G4 structures. The G4 structure is induced by both unprocessed NCp15 and mature NCp7, which indicates that both proteins may participate in genome recognition, recombination, dimerization and packaging. NCp7 could act through a potential mechanism that involves synaptic G4 intermediates, as illustrated in Figure 3 (115).

### Regulation of HIV-1 promoter activity

**Figure 4.**
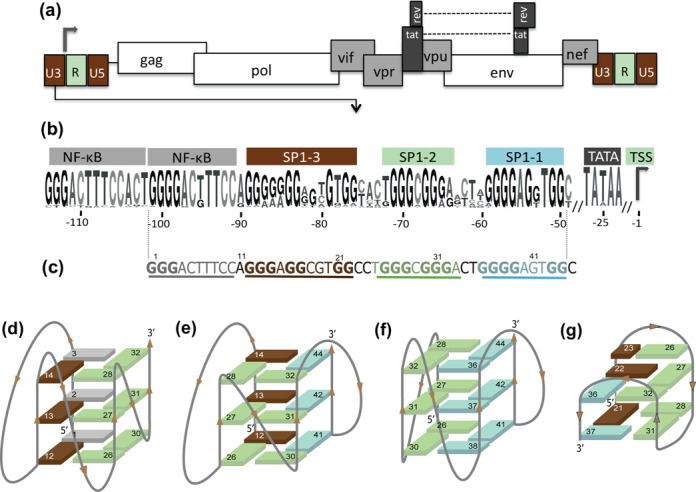
(**a**) Genomic structure of the HIV-1 provirus and (**b**) LOGO representation of the G-rich region for the HIV-1 promoter generated using the weblogo software (183) and based on an alignment of 1684 HIV-1 sequences provided by the HIV-1 database (www.hiv.lanl.gov). (**c**) The U3 region of the LTR from the HXB2_LAI;NC_001802 HIV-1 representative strain. (**d**, **e** and **g**) Topologies of the LTR G4s determined using Clerocidin and DMS-mediated footprinting assays (116) for the (d) 1–32 nt segment, (e) 12–44 nt segment and (**f**) 26–44 nt segment. (g) Topology of the 12–37 nt segments determined using nuclear magnetic resonance (NMR) (117).

Transcription of the viral DNA is performed by the cellular RNA polymerase II from the viral promoter located in the 5′-Long terminal repeat (LTR) of the proviral genome. The U3 region (Figure 4a) contains a G-rich sequence 50 nucleotides upstream from the transcription-starting site (TSS) and close to the TATA box (Figure 4b). This sequence overlaps with the so-called minimum promoter, which is composed of three SP1 as well as two NF-kB binding sites and is crucial for transcription initiation. The presence of eight blocks of guanines suggests that this region is a good candidate for G4 formation.

Two independent biophysical studies based on DMS-mediated foot-printing assays or NMR recently evaluated the ability of this 45-nt G-rich sequence to form G4 structures (116,117). DNA fragments spanning this region (corresponding to two or three SP1 or NF-kB binding sites with oligonucleotides ranging from 19 to 32 bases) were all able to form stable parallel and anti-parallel G4 topologies with melting temperatures ranging from 43°C to 56°C in 100-mM KCl solutions. The three models are formed with rather long sequences (up to 32 nucleotides), allowing the formation of long loops of 5 to 12 nucleotides (Figure 4 d, e and f). Interestingly, a G4 structure was also observed in the NMR study with an anti-parallel G4 core composed of only two tetrads and an additional Watson–Crick base pair that stacks on top of the upper tetrad (Figure 4g). Notably, these four topologies are mutually exclusive, and forming one of these G4s in the promoter will prevent formation of the three alternative conformations. Thus, the equilibrium between these forms may play a role in regulating promoter activity.

Recently, the interaction between the SP1 protein and a fragment of the HIV-1 promoter sequence folded into a G4 was studied (107). Piekna-Przybylska *et al.* used an affinity-based selection approach using biotinylated G4s immobilized on streptavidin-coated magnetic beads. The pull down experiments followed by western blotting revealed that the SP1 protein can bind the HIV-1 promoter sequence when it adopts a G4 conformation. Perrone *et al.* analyzed the effect of point mutations that disrupt the G4 structures formed in the promoter (116). The wild-type (WT) and mutated LTR promoters were cloned upstream of a firefly gene in a promoter-free plasmid. In HEK 293T cells, the promoter activity of the mutated sequences (unable to form G4) was twice as high as the WT LTR. These data suggest that G4s act as repressor elements in the transcriptional activation of HIV-1. Therefore, G4 structures might be critical for HIV-1 fitness and represent novel targets for antiviral drug development (see below). A better understanding of the role for G4s in regulating the HIV-1 promoter activity would also shed light on HIV-1 latency and reactivation mechanisms, from which a new landscape may emerge in clinical research to eradicate HIV from reservoirs (118).

### G4s and HIV-1 proteins expression

A G-rich sequence composed of three conserved clusters has been identified in the reading frame of the negative regulatory factor (Nef) (119). This coding sequence is located at the 3′-end of the genome and overlaps with the 3′-LTR (Figure 4a). Isolated nef G-stretches can form G4s *in vitro*. Moreover, their stabilization represses Nef expression and decreases viral replication. Nef has multiple roles during HIV infection and has been implicated in immune evasion by promoting CD4 and major histocompatibility complex (MHC) molecule down-regulation. Importantly, the absence of Nef seems to correlate with low viral load and inhibition of disease progression. Thus, targeting the G4s located in the Nef coding sequence is a new attractive therapeutic opportunity.

### Simian virus 40 (SV40)

SV40 is a polyomavirus with a 5-kb, closed circular and double-stranded DNA genome that codes for six proteins and includes a non-coding regulatory region (NCRR). This latter regulatory region contains the ORI and the encapsidation sequence (*ses*) but also controls the transcription direction (early versus late transcription). Notably, six GC boxes (GGGCGG) are present in this region, which can form an unusual quadruplex structure containing a C-tetrad stacked between two G-tetrads as determined by NMR (120). These repeated sequences are binding motifs for SP1 and, therefore, play an important role in early transcription. On the other hand, SV40 genome replication requires the large T-antigen (TAg), which is a multifunctional protein that binds the ORI, has adenosine triphosphate-dependent helicase activity and interacts with cellular proteins such as p53 and Rb (121). Interestingly, TAg can unwind G4 DNA structures (122,123); thus, it might play a crucial role in regulating replication as well as early and late transcription. Perylene di-imide derivatives (PDI) stabilize G4 structures and inhibit both the G4 and TAg duplex DNA helicase activities. Hence, PDI provide tools for probing the role of the G4 helicase activity in SV40 replication (123,124) and introduces new insights into the link between helicases and tumorigenesis (125) or other human genetic diseases (126).

### Human papillomaviruses

The human papillomaviruses (HPV) family consists of more than 120 viruses; approximately half are sexually transmissible and 15 are considered high risk due to their carcinogenic properties. These viruses are one of the most common sexually transmitted infections and induce cervical cancer. Notably, HPV16 and HPV18 contribute to 70% of cervical cancer induced by HPV infections. The HPV genome consists of 8 kb, circular, double-stranded DNA and integration into the infected cell induces dramatic genome instability (127). The open reading frames encode six **‘**early**’** proteins (E1, E2, E4, E5, E6 and E7) and two **‘**late**’** structural proteins (L1 and L2). The late proteins are major and minor capsids, respectively, and association with 72 L1/L2 heterodimers forms star-shaped capsomeres. However, L1 can self-assemble into 55–60 nanometers, virus-like particles, which can be used in prophylactic HPV vaccines to protect against an initial HPV infection.

G-rich regions have been found in HPV genomes, and their potential to fold into a G4 structure has been described (128). G-rich loci that fulfill the criteria for G4 formation have only been found in eight types of HPV. However, a strong argument for the relevance of G4s in HPV biology is that the viral protein E1 is a helicase that resembles SV40 TAg (121). Consequently, E1 may also present a G4 unwinding activity. For HPV52 and HPV58, potential G4-forming sequences are located in the long control region (LCR), which is a regulatory sequence composed of nearly 1 kb, suggesting a potential role in transcription and replication. The presence of G4s in the sequence coding for the L2 protein (HPV57), E1 (HPV32, HPV42) and E4 (HPV3, HPV9, HPV25) suggests that G4 formation may also alter alternative splicing necessary for producing viral proteins from the overlapping Open reading frame (ORFs). Targeting these G4s could potentially serve as a basis for novel antiviral therapies.

### Epstein–Barr virus

Also referred to as human herpesvirus 4 (HHV-4), Epstein–Barr virus (EBV) is a virus of the herpes family that can induce infectious mononucleosis and cancers, such as Hodgkin's lymphoma, Burkitt's lymphoma and nasopharyngeal carcinoma (129). In the case of HIV-1 co-infection, EBV is also associated with hairy leukoplakia and central nervous system lymphoma. The virus is ∼120 to 180 nm in diameter and is composed of a 172 kb DNA double helix that circularizes upon entry into the nucleus and becomes a viral episome. Human herpes viruses are mostly asymptomatic due to latency.

The Epstein–Barr virus encodes a genome maintenance protein (nuclear antigen 1, EBNA1), expressed in all EBV-associated malignancies. EBNA1 binds G-rich sequences at the viral replication origin, recruits the replication complex and is involved in metaphase chromosome attachment, which insures maintenance throughout mitosis (130). Thus, formation of secondary structures, such as G4s, may play a role in regulating EBV replication.

The level of EBNA1 synthesized is tightly controlled; it is sufficiently high to maintain viral infection but sufficiently low to avoid immune recognition by the host's virus-specific T cells. Regulation occurs during translation due to secondary structures present in the mRNA (131). Destabilization of the G4-forming sequence through antisense oligonucleotide annealing increases the translation rate and, consequently, promotes antigen presentation. On the other hand, stabilization of the G4 with a G4 ligand (pyridostatin) decreases EBNA1 synthesis and allows immune evasion. Interestingly, G4s are similarly observed in mRNA for other maintenance genes in the gammaherpesvirus family. These findings suggest that this mode of translational regulation may be more general among proteins that self-regulate synthesis. In addition, one could imagine alternative therapeutic strategies focused on targeting RNA structures within viral ORFs to interfere with the virus cycle as well as to promote antigen presentation and to stimulate the host immune response.

### Targeting viral G4s

Recently, a G4 ligand called quarfloxin (CX-3543) entered phase II clinical trials. It is capable to suppress MYC transcription by inhibiting the interaction between nucleolin and the c-myc G4 motifs (132). Even if CX-3543 failed trials because of bioavailability issues, original anti-cancer applications based on such ligands seem underway (133). Taken together, all these studies suggest that targeting these G4s could potentially serve as a basis for novel antiviral therapies. Hence, as described for cellular targets (134), small ligands that can stabilize the G4 structure may compose a potential new class of therapeutic agents to fight viral infections (62,135) [for reviews, see (136,137)]. Recent studies showed that G4 ligands, such as BRACO-19 and Tmpyp4, inhibited HIV-1 replication (116,119). Exhaustive viral assays demonstrated that BRACO-19 acts at both reverse-transcription and post-integration steps by targeting of the viral promoter (138).

Several laboratories are already working to identify G4 ligands that preferentially interact with the pathogen's G4 over cellular G4s. This new antiviral strategy presents many advantages: (i) these compounds target viral DNAs and RNAs, which are the source of the disease; (ii) high conservation of these targets across subspecies suggests that they are important for viruses and (iii) mutations will likely impact viral fitness, limiting the emergence of resistant strains.

## G4S AS ANTIVIRAL AGENTS

Nucleic acids have already been validated as therapeutics (139). The first example is Formivirsen (Vitravene^®^), a phosphorothioate anti-sense oligonucleotide approved from 1998 to 2002 to treat retinitis caused by Cytomegalovirus (CMV) (140). The second is Pegaptanib (Macugen^®^), used in the clinic from 2004 to 2011. This 2′-fluoropyrimidine RNA-based aptamer targets VEGF to treat neovascular age-related macular degeneration (141). However, those two drugs have been withdrawn due to the insufficient benefice associated with their use. Regarding G4s, certain G4 oligonucleotides present therapeutic potential, including the thrombin-binding aptamer (TBA, G_2_T_2_G_2_TGTG_2_T_2_G_2_) (142,143). The most promising G4 to date is AS1411 (AGRO100), a 26 bases oligonucleotide targeting nucleolin with anti-proliferative properties (144). AS1411 recently completed phase II clinical trials as anti-cancer drug (NCT00740441) with low toxicity, highlighting the high therapeutic potential of G4 (145).

For most applications, G4-forming molecules have been selected through combinatorial methods (e.g. pegaptanib, anti-TBA). Two key protocols have been developed. First, polynucleotide arrays facilitated rapid detection of molecules with an affinity to the target. Then, the development of SELEX (systematic evolution of ligands by exponential enrichment) facilitates isolation of oligonucleotide sequences with the capacity to recognize virtually any class of target molecule with high affinity and specificity (146). These oligonucleotides, ‘aptamers’, are emerging as a class of molecules that rival antibodies in both therapeutic and diagnostic applications (147,148). In this section, we describe a few examples of aptamers specifically selected to interact with viral components and interfere with viral replication.

### Hepatitis A virus

Hepatitis A virus (HAV) belongs to the *picornaviridae* family. Even without a specific treatment for HAV infection, a vaccine has been produced to protect against initial infection. However, only a limited number of countries recommend vaccination because HAV is rarely lethal. The HAV genome comprises a 7.5-kb single-stranded (+) RNA with a 5′-UTR and 3′ poly(A) tail. It encodes for a single polyprotein, which includes the 3C protease. This enzyme is crucial for the virus because it cleaves the polyprotein into several capsid proteins and non-structural proteins. Additionally, the 3C protease binds regulatory structural elements at the 5′-UTR, which control viral genome replication. Thus, the 3C protease is an attractive target for develop a specific antiviral treatment. Recently, hexadeoxyribonucleotides that specifically bind the HAV 3C protease were identified through a hexanucleotide array (149). *In vitro* experiments showed that the hexanucleotide GGGGGT (G_5_T) forms a tetramolecular G4, binds the C-terminal domain of HAV protease and is a potent protease inhibitor (150).

### Influenza virus (the flu)

Every year in winter, influenza viruses cause seasonal respiratory disease epidemics (151). Three subtypes have been defined (A, B and C), which depend on the surface proteins (hemagglutinin and neuraminidase). Sixteen hemagglutinin (H) and nine neuraminidase (N) variants have been discovered, but only H 1, 2 and 3 as well as N 1 and 2 are commonly found in humans. These viruses belong to the *Orthomyxoviridae* family, which composes enveloped viruses with a single-stranded negative-sense RNA. The genome is 10 to 15 kb but is segmented into multiple (7 or 8) molecules. Each RNA is 0.9 to 2.3 kb and encodes for 1 or 2 proteins. The non-structural protein 1 (NS1) is a 26 kDa multifunctional protein and participates in protein–protein and protein–RNA interactions (152). During infection, NS1 interferes with cellular mRNA biology (splicing, maturation and translation). As a result, NS1 prevents interferon (IFN) production, which leads to inhibition of the host's innate immunity (153). After 15 cycles of SELEX, which employs a 45-base variable sequence, a G4-forming aptamer was selected that presents high affinity for the NS1 RNA binding domain (Kd ≈ 20 nM) (154). In a cellular context, this G4 can block NS1 and restore INF production, which results in antiviral activity without cellular toxicity.

### Severe acute respiratory syndrome coronavirus (SARS-CoV)

Severe acute respiratory syndrome coronavirus (SARS-CoV) is an enveloped virus with a single-stranded RNA genome ∼30 kb long. It encodes 2 poly-cistronic ORFs, which code for 16 non-structural proteins (Nsp). Since the 2003 outbreak, the three-dimensional structures of several key proteins have been determined (e.g. RDRP, 3C-like protease). The non-structural protein 3 (Nsp3) is one component of the viral replicase complex and contains a domain referred to as SARS unique domain (SUD), which interacts with G4s. Thus, G4s are also relevant for the coronavirus and might be involved either in viral replication or host immune evasion (155). On the other hand, using a SELEX approach with oligonucleotides that harbor a 30 nucleotide variable sequence and following 20 rounds of selection, DNA aptamers against the SARS-CoV helicase were isolated (156). These aptamers compose two distinct classes, G4 and non-G4 forming sequences, which were determined through circular dichroism and gel electrophoresis. Their inhibitory effect on viral replication is being studied with encouraging results.

### Human immunodeficiency virus

Combinatorial approaches have been used to identify several aptamers that target HIV-1. The first RNA aptamer isolated using the SELEX approach was an RNA pseudoknot inhibitor of HIV-1 RT (157). Using another procedure, the SURF (synthetic unrandomization of randomized fragments) (158), ISIS 5320 was selected and exhibited sub-micromolar inhibition of HIV-1. ISIS 5320 is a phosphorothioate containing DNA octamer (T_2_G_4_T_2_) that forms G4 with anti-HIV properties. More specifically, it inhibited cell-to-cell and virus-to-cell spread of the HIV-1 by interacting with the V3 loop located on the viral glycoprotein gp120 (159–161). Its G4 structure and phosphorothioate backbone were reported as essential to this inhibition. Later studies showed that phosphodiester oligonucleotides containing only G and T inhibit HIV-1 replication and the most potent molecule, GTG_2_TG_3_TG_3_TG_3_T (T30177, EC_50_ at ∼100 nM), formed a G4 *in vitro* (162–164). This oligonucleotide and related molecules, such as T30923 (G_3_TG_3_TG_3_TG_3_T, Figure 1j), are potent HIV-1 IN inhibitors *in vitro*. T30177 was the first IN inhibitor tested in clinical trials (Zintevir™ developed by Aronex Pharmaceuticals in 1996) (165). However, the action mechanism in cells is more complex because it also targets viral entry (166).

In attempts to obtain natural-type oligonucleotide, Hotoda *et* *al.* identified a hexamer (TG_3_AG) also targeting HIV-1 entry through gp120 binding (167). This ‘Hotoda's sequence’ adopts a tetramolecular G4 structure and submicromolar HIV-1 inhibition was described for derivatives with 5′-end substitutions. Once more, the antiviral activity of the molecule was directly linked to its capability to form G4s.

Later, SELEX approaches were developed to isolate DNA aptamers with high affinity for the RNase H domain of HIV-1 RT. Thus, the target protein used was either the isolated RNase H domain (p15) or the functional heterodimer p66/p51 with a counter-selection that used the p51/p51 form (truncated for the RNase H domain) (168,169). Interestingly, these selections mainly led to aptamers with G-rich sequences capable of forming G4s. Some, but not all, of these G4 inhibited the RNase H activity of HIV-1 RT *in vitro* with an IC_50_ in the nM range (168). Surprisingly, these G4 aptamers (93del and 112del) were also potent IN inhibitors *in vitro* with IC_50_ values in the range 10–40 nM (170). This dual inhibition can be explained by the structural similarities between the IN active site and RT RNase H domain (171). The aptamer 93del can form an original dimeric interlocked G4 (Figure 1i), which is stable even at temperatures over 90°C (172). Through comparing the structures of the G4-forming aptamers that inhibit IN, IN inhibition likely requires a stack of 6 G-tetrads (164).

Similar to T30177, 93del is a potent antiviral agent with multimodal inhibition and, in cells, targets the viral entry step, reverse transcription and integration (173). Additional *in vitro* studies indicated that free 93del and T30923 can enter human cells, including epithelial (HeLa), hepatic (Huh7) and lymphocytes (H9) cells (174). However, striking differences were observed in the presence of viral particles; HIV-1 strongly stimulates cellular uptake of aptamer (174). This latter observation opens an opportunity for specific drug delivery to cells that are infected, which may prevent intracellular side effects from G4 off-targeting.

## G4S AS DETECTION TOOLS

Given the remarkable physical properties of G4s, scientists have engineered innovative tools using G4 folding to control ribozyme activity and constructed probes for state-of-the-art imagery as well as quantification techniques, among other approaches. As an example, the aforementioned G4 93del was engineered into aptamer beacons to visualize endogenous protein HIV-1 reverse transcriptase in living cells (175). The following section describes two such innovations related to viruses.

### Hepatitis delta virus (HDV)

HDV is a small, enveloped virus that depends on hepatitis B virus (HBV) for propagation (176). Its genome is a circular, single-stranded (−) RNA molecule of 1679 nucleotides. However, due to self-complementarity over ∼70% of its length, it folds into a partially double-stranded molecule (rod-like RNA structure). Because HDV does not encode for a polymerase, replication of its genome relies on cellular enzymes. Upon polymerization following the ‘double rolling circle’ mechanism, genomic and anti-genomic circular RNA is produced with linear polyadenylated mRNA that only codes for the HDV protein, delta antigen (HDAg). However, HDV also encompasses an 85 nucleotide ribozyme with self-cleavage activity. Molecular engineering led to development of a ‘G-quartzyme’, which is a ribozyme controlled by a G4 structure (177,178). Stabilization of the G4 at the 3′ end by monovalent ions (K^+^) activates the ribozyme.

### Cauliflower mosaic virus (CaMV)

Cauliflower mosaic virus (CaMV) is a pararetrovirus that infects plants (179). Its genome is an 8-kb, circular, double-stranded DNA molecule produced through reverse transcription of a pre-genomic mRNA. Therefore, transcription of the genome is a crucial step for two reasons: (i) it is necessary to code for several viral proteins and (ii) it generates 35S RNA that is slightly longer than the genome (terminally redundant), which serves as a matrix to replicate the DNA genome. Because the unique CaMV promoter, the 35S promoter, is a strong and constitutive promoter, it has been widely used to develop genetically modified organisms (GMOs). Consequently, there is a growing need to detect GMOs in the food industry, and many methods have been developed based on ELISA, polymerase chain reaction and other techniques. Notably, a fluorescent assay has been engineered based on G4 formation. It involves two specific primers that recognize the 35S promoter. Each primer is linked to a double repeat of the human telomeric sequence TTAGGG. As a consequence, binding the two primers facilitates inter-strand quadruplex formation. Upon adding berberine (a selective G4-binder), a strong fluorescent signal is produced (180), which facilitates promoter detection.

## CONCLUSION

The X-ray structure of G-tetrads was determined in the early 60s; in the 90s, G4s remained an intriguing deviation from the Watson–Crick canonical structure. However, a remarkable quantity of information has been obtained over the past two decades, which has allowed this field to grow from basic science to clinical application (181,182). Thus, the presence of G4s in telomeres and oncogenic promoters has opened broad opportunities for understanding and generating new treatments against cancer.

The viral world is extensive and includes viruses with replication schemes based on RNA (e.g., flaviviruses), DNA (e.g., papillomavirus) or intermediates thereof (e.g., retroviruses) and G4s are part of their life cycle. First, they must address the presence of G4s in their hosts, and second, they must also contain G4-forming sequences that regulate important replication steps. Several challenges remain to better define the features that provide specific G4 motifs with the ability to function as structural elements. Nevertheless, G4 sequences and G4-binders have been identified in some of the most pathogenic viruses; thus, they are attractive targets for controlling viral infections.

## ACCESSION NUMBERS

PDB IDs: 2KF8, 2LPW, 2M4P, 1Y8D and 2LE6.
